# Asymmetric Cell Division and Template DNA Co-Segregation in Cancer Stem Cells

**DOI:** 10.3389/fonc.2014.00226

**Published:** 2014-08-21

**Authors:** Sharon R. Pine, Wenyu Liu

**Affiliations:** ^1^Department of Medicine, Robert Wood Johnson Medical School, Rutgers Cancer Institute of New Jersey, Rutgers, The State University of New Jersey, New Brunswick, NJ, USA

**Keywords:** asymmetric cell division, immortal DNA, co-segregation of template DNA, p53, cancer stem cells, Wnt, Notch

## Abstract

During tissue homeostasis, normal stem cells self-renew and repopulate the diverse cell types found within the tissue via a series of carefully controlled symmetric and asymmetric cell divisions (ACDs). The notion that solid tumors comprise a subset of cancer stem cells (CSCs) with dysregulated self-renewal and excessive symmetric cell divisions has led to numerous studies aimed to elucidate the mechanisms regulating ACD under steady-state conditions, during stem-cell expansion and in cancer. In this perspective, we focus on a type of asymmetry that can be established during ACD, called non-random co-segregation of template DNA, which has been identified across numerous species, cell types, and cancers. We discuss the role of p53 loss in maintaining self-renewal in both normal and malignant cells. We then review our current knowledge of the mechanisms underlying co-segregation of template DNA strands and the stem-cell pathways associated with it in normal and CSCs.

## Introduction

### Tissue homeostasis

The continual maintenance of a reservoir of tissue-specific stem cells affords an organism with the ability to generate all the differentiated cells needed for tissue homeostasis and repair throughout its lifespan. During homeostasis or repair, a stem cell can undergo asymmetric cell division (ACD) to simultaneously generate daughters with differing cell fates. One daughter remains a stem cell and the other gives rise to differentiated progeny that carry out the functions of the mature tissue. When a stem cell asymmetrically divides, it does so by actively segregating one or more intrinsic cell fate-determining constituents, or by polarizing the cell cortex and aligning the mitotic spindle so that the two daughter cells are exposed to differing external stimuli directing their cell fate potential (1, 2). In certain circumstances, such as during development or after stem cell depletion from excessive injury, normal stem cells can also be exponentially expanded through a series of symmetric divisions. Thus, both asymmetric and symmetric cell divisions can lead to stem cell self-renewal.

### Asymmetric cell division in cancer

**Figure 1:**
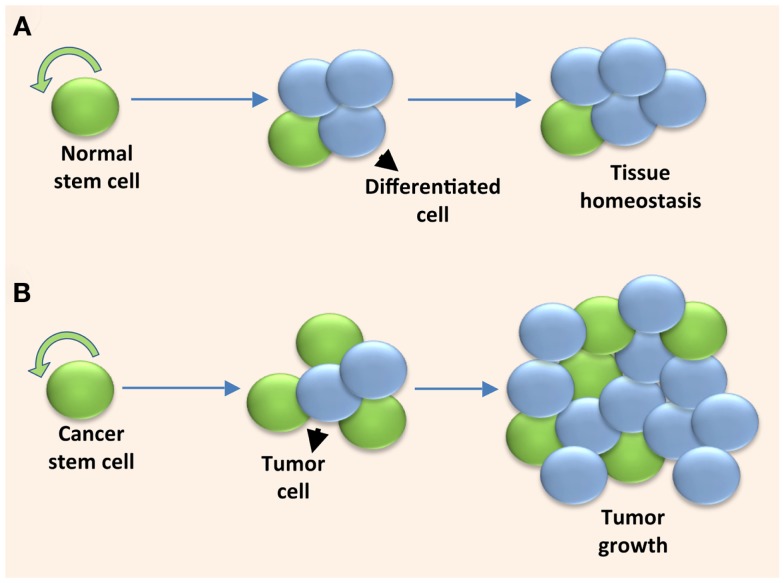
**Solid tumors are fueled by cancer stem cells undergoing excessive symmetric cell divisions**. **(A)** A normal stem cell maintains tissue homeostasis by self-renewing and providing the differentiated cell types within the tissue through a controlled series of symmetric and asymmetric cell divisions. **(B)** A cancer stem cell fuels uncontrolled tumor growth when it undergoes excessive symmetric cell divisions due to dysregulated self-renewal.

It is necessary to maintain a tightly controlled balance between symmetric and asymmetric stem cell divisions in order to preserve an optimal number of stem cells within a tissue or organ. When the balance is shifted to favor excessive symmetric self-renewing divisions, it can lead to a hyperplastic state and cancer development [reviewed in Ref. (3)]. The tumor itself, however, can be viewed as an abnormal organ in which the mature tumor cells are seeded in a hierarchical fashion by a stem cell-like population, called cancer stem cells (CSCs). Unlike normal stem cells, CSCs have lost the ability to control their mode of cell division, resulting in continual excessive symmetric cell divisions and consequential uncontrolled tumor growth (Figure 1).

A debated question in CSC biology is whether neoplastic transformation emerges from normal stem/progenitor cells or from more differentiated cells that commandeer stem cell properties during or after the oncogenic process. Since normal stem cells inherently possess many of the properties that CSCs exploit, it seems more likely that a tumor would arise from a normal stem cell. Lineage tracing experiments and genetically engineered mouse models in which oncogenic events were restricted to specific cell types have confirmed that transformation can indeed arise from the normal stem cell population (4). However, several reports have demonstrated that certain progenitor and differentiated cells can also be transformed [reviewed in Ref. (5)]. Thus, tumors comprise a hierarchically organized cell population seeded by CSCs regardless of the cell of origin. Notwithstanding, uncovering the mechanisms regulating normal stem cell self-renewing divisions could provide insights into how those mechanisms are disrupted in CSC biology, and lead to pharmacological strategies to deplete the CSC pool altogether.

Another intriguing feature in stem cell biology is cellular plasticity. It was long believed that tissues were hierarchically organized in which the tissue-specific stem cells reside at the apex and differentiation could only occur in one direction. However, recent *in vivo* lineage tracing experiments have proved that differentiated cells within a tissue can replenish the stem cell population (6, 7), especially after an event that induces stem-cell depletion. Our new understanding of normal cellular plasticity lends credence to the argument that cancer cells can dedifferentiate into CSCs. A normal stem cell or CSC that arises from a more differentiated cell would theoretically repossess the ability to symmetrically or asymmetrically divide. How cellular plasticity might affect genomic integrity, proliferative lifespan of the resultant stem cell, or organismal aging is unknown.

## Co-Segregation of Template DNA Strands

One of the most intriguing types of asymmetry during ACD is the active segregation of the “older” template DNA strands specifically to one daughter cell and the shunting of the newly synthesized DNA strands to the other. As elegantly recounted by K. Gordon Lark in the research topic on stem cell genetic fidelity (8), the discovery of template DNA strand co-segregation during cell division resulted from a series of experiments in the mid to late 1960s (9). Even though it has been observed across numerous species and tissue types, the notion of template DNA co-segregation has been debated extensively because of a failure to observe it in some tissue stem cells, which might have been caused in some cases by the differing methods utilized to detect it [reviewed in Ref. (10)]. The initial observations by Lark led to theories explaining why cells would actively sort their DNA into old and new strands during cell division. One explanation, called the “immortal DNA strand hypothesis,” states that stem cells prevent cancer-causing replication-induced DNA mutations by exclusively co-segregating their older, “immortal” DNA strands to the long-lived daughter stem cells and passing their newly synthesized DNA off to the daughter cells that give rise to differentiated cells (11).

An alternative though not mutually exclusive hypothesis explains that the older template DNA strands harbor epigenetic marks that differ from those on the newer DNA strands. The differing epigenetic tags direct differential gene expression after cell division, leading to differing fates of the daughter cells. If the older versus newer sets of sister chromatids harbor differing epigenetic marks, it is likely that the tags are added during DNA synthesis, though this has not been definitively tested (12). The most convincing evidence supporting this hypothesis is from Amar Klar’s group, in which they demonstrated that specific sister chromatids are segregated during ACD in *Caenorhabditis elegans* and mice (13–15), which was based from their studies performed on the fission yeast *Schizosaccharomyces pombe*. Yeast sister cells developmentally differed by inheriting sister chromatids that were differentiated by epigenetic differences [reviewed in Ref. (16)]. While neither the immortal strand hypothesis nor epigenetic changes have been proven to be the driving functional consequence of co-segregation of template DNA strands in multicellular organisms; preservation of genomic integrity and gene expression patterns prior to stem cell division would both be beneficial to stem cell and organismal survival.

### Template DNA strand co-segregation in cancer

In addition to normal tissues, we and others have found evidence for template DNA strand co-segregation in cell lines and short-term cultures of human tumors from numerous cancer types (17–22). It is unknown if the retention of template DNA co-segregation in cancer cells is a passive, albeit dysregulated, remnant of hierarchical organization in normal tissues, or if it offers a survival advantage. The mutation theory of the immortal DNA strand hypothesis is far more relevant in normal stem cells compared to malignant cells, because genomic instability and consequential tumor progression would be favored for tumor cell survival. But epigenetic regulation plays a central role in tumor progression (23), so priming the daughter cells with an epigenetic signature prior to cell division could potentially accelerate tumor progression.

We reported that the frequency of template DNA co-segregation in lung and breast cancer cell lines decreases when the microenvironment favors symmetric self-renewal (17, 18), and that the template DNA strands are inherited by the daughter cell with more CSC-like qualities (17). Therefore, it is likely that the mechanism underlying the decision to self-renew is an active process at least partly under the control of extrinsic factors. Elucidation of the molecular mechanisms driving template DNA strand co-segregation in cancer would provide important clues for why and how CSCs undergo excessive self-renewal.

## p53 in Asymmetric Cell Division and Template DNA Strand Co-Segregation

### Loss of p53 during stem cell self-renewal

p53 is the most extensively studied tumor suppressor gene and is often referred to as the “guardian of the genome” (24). The best understood functions of p53 are in the orchestration of cellular responses to different stress stimuli through the induction of cell cycle arrest, DNA repair, senescence, and apoptosis. Recently, p53 was found to function in the maintenance of a stem cell state. Work that has emerged from reprograming somatic cells into pluripotent cells, referred to as induced pluripotent stem cells (iPSCs), has confirmed the role of the p53 pathway in the production of this type of stem cell. Loss or knockdown of p53 not only increases reprograming efficiency, it also accelerates the kinetics of reprograming [reviewed in Ref. (25)], though it is not yet clear exactly how loss of p53 contributes to improved reprograming efficiency. There has been mounting evidence that loss of p53 contributes to normal stem cell self-renewal, especially after DNA damage. An excellent example supporting this comes from work on mammary epithelial and hematopoietic stem cells. Pier Pelicci’s group demonstrated that unlike progenitor and differentiated cells that activate p53 in response to DNA damage, p53 is not activated in normal stem cells. Intriguingly, p53-independent upregulation of p21 in normal stem cells after DNA damage inhibits p53 activity and shifts the cell divisions from asymmetric to symmetric self-renewal (26). Given the central role of p53 in normal stem cell self-renewal, and the conjecture that cancer is a disease of excessive self-renewal, it stands to reason that p53 mutations may induce excessive self-renewal of CSCs. Loss of p53 resulted in the expansion of pre-malignant mammary stem cells through increased symmetric self-renewing divisions, and when p53 was reactivated, there was a reduction in the CSC pool due to restoration of ACDs (27). Thus p53 likely plays a direct and central role in regulating the switch between asymmetric and symmetric cell divisions of both normal stem cells and CSCs.

### p53 in asymmetric cell division

Sherley’s group demonstrated that, in cell lines derived from murine embryonic fibroblasts and mammary epithelial cells, expression of wildtype p53 at physiological levels shifted the balance from symmetric to asymmetric stem cell kinetics. In this system, asymmetric self-renewal was defined as a cell giving rise to a stem-like cycling cell and a non-dividing daughter cell (28). Expression of p53 also altered co-segregation of template DNA. Under conditions that favored symmetric self-renewal in which p53 was not expressed, chromosomes were randomly segregated during cell division. In contrast, expression of p53, which favored asymmetric self-renewal, induced a shift toward increased co-segregation of template DNA strands (28). Thus, p53 induces asymmetric self-renewal of adult stem cells not only at a functional but also at a genomic level. These data support the notion that asymmetric self-renewal and template DNA strand co-segregation are at least sometimes coupled in stem cells.

### The role of DNA damage in asymmetric cell division

After a tissue damaging event, a large number of stem cells would be needed to quickly respond, expand, and replace damaged cells. However, cells could be most vulnerable to DNA replication errors during hyperproliferation and tissue regeneration. The fact that the genome would actively symmetrically segregate its damaged DNA to both daughter stem cells during rapid expansion seems counter-intuitive. According to the “immortal” DNA strand hypothesis, stem cells avoid passing their nascent DNA strands, wrought with potential DNA replication errors, off to its daughter stem cell while co-segregating its older and presumably undamaged DNA to its daughter stem cell. Why then would stem cells symmetrically self-renew and risk passing replication errors off to the daughter stem cells during a time of rapid cell cycling? An assumption of the “immortal” DNA strand hypothesis is that stem cell DNA segregation is under normal steady-state conditions. Perhaps when faced with a DNA damaging event and expansion of the stem cell pool is necessary to regenerate lost cells, the symmetrically self-renewing stem cells employ an as yet undiscovered DNA damage response pathway unique to repairing the stem cell genome. Based on the work from Pelicci’s group as described above, the pathway is likely to be independent of p53. Furthermore, mouse embryonic stem cells (ESCs) undergo repair double strand breaks much faster than differentiated cells, primarily using homologous recombination [reviewed in Ref. (29)]. Given that reduction of p53 supports replication-associated homologous recombination, it is possible that this DNA repair pathway prevails in symmetrically dividing somatic stem cells, though this would need to be tested. A stem cell-associated DNA repair pathway would also complement Cairn’s original observations in which he found a mathematical discrepancy between predicted DNA mutation rates in human tissue cells and human cancer incidence (11). Furthermore, it was proposed that chemical modifications or DNA mutations in the “immortal” stem cell genome contributes to aging (30). Together with co-segregation of template DNA strands, such a DNA repair mechanism during symmetric self-renewal would protect the stem cell genome from not only DNA replication errors but also DNA damage, and possibly even the cellular aging process.

### p53 isoforms

We would be remiss to discuss the central role of p53 in stem cell ACD without mentioning p53 isoforms. The human TP53 gene encodes at least 13 isoforms that are the result of alternative splicing or alternative promoters (31). Two of the best studied human p53 isoforms functionally interact with full-length p53. Isoform Δ133p53 is an N-terminally truncated isoform that inhibits full-length p53 in a dominant-negative manner. Isoform p53β is a C-terminally truncated isoform that cooperates with full-length p53 (32). Curtis Harris’ group, with whom Sharon R. Pine had her post-doctoral fellowship training, demonstrated that p53 isoform switching can modulate the youthfulness of a cell. They showed that p53β represses and Δ133p53 increases the replicative lifespan of normal human fibroblasts cultured *in vitro*. Furthermore, p53 isoform switching was associated with tumor progression in colon cancer (33), consistent with the modes of functional interaction between these p53 isoforms and full-length p53. They further demonstrated that p53 isoform switching directly modulates cellular replicative lifespan, senescence, and activation of CD8^+^ T lymphocytes *in vivo* (34). An untested question to date is whether p53 isoforms are associated with ACD. This could have important implications in cancer biology because p53 isoform expression patterns and activity would theoretically depend on the location of the TP53 mutation. We did not observe a correlation between p53 mutation or deletion status and frequency of template DNA asymmetric segregation in a panel of breast cancer cell lines (18). It would be intriguing to test if specific p53 isoforms are involved in the regulation ACD or co-segregation of template DNA strands.

## Molecular Mechanisms of Template DNA Strand Co-Segregation

Template DNA strand co-segregation has been observed in stem cells across numerous tissue types, but the underlying molecular mechanism(s) regulating this process has remained elusive. In order to decipher how template chromosomes are asymmetrically segregated during cell division, one must identify what “marks” the newer and older DNA strands, what cellular machinery recognizes those marks and finally, what process actively recruits the chromosomes bearing those marks to one daughter cell. The Tajbakhsh group speculated that the mother centrosome might anchor certain sister chromatids to the polarized cortex (35). How the kinetochore might relay information to the centrosome and cell cortex via the mitotic spindle to mediate asymmetric chromosome segregation is still unsolved.

Much of the effort to identify the molecular mechanisms responsible for a cell’s decision to co-segregate its template DNA strands has been pioneered by the Sherley group. Rambhatla et al. demonstrated that template DNA co-segregation was induced by p53 and required the down-regulation of an enzyme essential for guanine nucleotide biosynthesis, IMP dehydrogenase (36). Perhaps the mechanism by which p53 regulates template DNA strand co-segregation is linked to the concentration of guanine nucleotides, although it could also be linked to one or more of many other cellular functions regulated by guanine nucleotides, such as signal transduction, glycoprotein synthesis, or energy transfer. It was not clear if down-regulation of IMP dehydrogenases was sufficient for p53-induced template DNA co-segregation, or if one or more of the numerous p53-regulated genes were also key modulators of template DNA strand co-segregation.

Sherley’s group later discovered that the template DNA strands harbor more of the histone H2A variant H2A.Z that is “uncloaked”, meaning that it is more detectable by immunofluorescence (37). They more recently reported that the template DNA strands had a higher content of 5-hydroxymethylcytosine (5hmC) than the newer DNA strands, which is an intermediate during DNA methylation (38). Although 5hmC, or a unique protein complex that binds 5hmC in stem cells, could possibly be the elusive “mark” of template DNA strands, differences in DNA demethylation between the two daughter cells of an asymmetric stem cell division are also consistent with the idea that the template DNA strand co-segregation dictates cell fate via a differential regulation of gene expression in the two daughter cells. Klar’s group discovered additional clues that could underlie the mechanisms of template DNA co-segregation. They found that in *S. pombe* and *Schizosaccharomyces japonicas* yeast that a DNA strand-specific epigenetic imprint at the mating locus initiates a recombination event, which is required for cellular differentiation (16, 39). In their system, an inherent chirality of the double-helical structure of DNA was needed to achieve ACD. They later provided evidence for this during mouse development (13). An intriguing question is whether a similar epigenetic mechanism is conserved on a global level in multicellular organisms to dictate different cell fate potentials of the daughter cells.

### Wnt pathway

Additional clues about the mechanisms driving template DNA strand co-segregation have emerged from studies of stem-cell signaling pathways that are either associated with or actively affect the balance of ACD and template DNA strand co-segregation. Wnt signaling is a key stem cell signaling pathway implicated in self-renewal of both normal stem cells and CSCs, as well as epithelial–mesenchymal transition in cancer (40). Inhibition of Wnt signaling decreased the frequency of template DNA co-segregation in gastrointestinal cancer cell lines (21). This work was challenged in a separate study in which random segregation of template DNA in colorectal CSCs was induced by Wnt signaling (41). It would be interesting to test if Wnt inhibition or activation reduces template DNA co-segregation by increasing symmetric divisions of non-stem cells, or increasing symmetric self-renewing divisions within the stem cell population, respectively. Although it was uncertain whether Wnt signaling played a direct role in regulating template DNA co-segregation, or if one of the many pathways modulated by Wnt/β-catenin signaling were responsible, these studies demonstrated that factors in addition to p53 or IMP dehydrogenase may directly modulate co-segregation of template DNA, and particularly, genes within signaling pathways that direct stem cell fate.

### Notch pathway

The most extensively studied pathway associated with template DNA co-segregation is the Notch signaling pathway. Notch is a developmental and adult stem/progenitor cell transcription factor that regulates self-renewal and differentiation in a highly cell-type and context-specific manner (42). Notch signaling is dysregulated across numerous types of cancer and plays a key role in regulating CSC self-renewal (43). The Notch pathway directly participates in ACD across several cell types in lower organisms such as *Drosopila* and *C. elegans*, as well as in mice and human beings (1). For example, in *Drosophila* neuroblasts the Notch inhibitor Numb is inherited by the daughter cell that is destined to undergo differentiation (1, 44). When Numb is mutated, the neuroblasts hyperproliferate and form a tumor-like phenotype (45, 46). Notch dysregulation is also an active participant during the development of cancer in mouse models (47–49), though whether the role of Notch in carcinogenesis involves shifting the mode of CSC divisions toward excessive symmetric self-renewal has not been established.

Notch signaling has been linked to template DNA co-segregation. One example has been demonstrated in muscle satellite stem cells. Satellite cells self-renew and provide the generation of myoblasts that are needed for skeletal muscle homeostasis and repair. Muscle satellite stem cells co-segregate their template DNA strands (50, 51) and the daughter stem cells that inherit the template DNA preferentially inherit Numb (50). In colon CSCs, an increase in Notch mRNA levels caused by knockdown of miR34a increases symmetric self-renewing divisions and decreases ACDs (52). It is still unclear whether Notch signaling is a cellular constituent that is asymmetrically segregated in parallel with but independent from template strand co-segregation, or if Notch signaling is a regulator or effector of template DNA co-segregation.

## Conclusion

It has been nearly 50 years since the first discovery of template DNA co-segregation. Though it has been observed across numerous species, normal tissue, and cancer, we have still only scratched the surface toward elucidating the purpose for conserving template DNA strands in one daughter cell and the mechanisms regulating the process. Consistent with the notion that the immortal DNA strand hypothesis does not fully capture why stem cells asymmetrically segregate their template DNA, there has been increasing evidence that the mother cell controls gene expression in the daughter cells through asymmetric segregation of epigenetic marks. With recent advances in genomics, chromosome-imaging technologies, and genetically modified model organisms [reviewed in Ref. (10)], the outlook for success in further answering these daunting questions is bright. Elucidating why and how template DNA strands are co-segregated is a fundamental aim in basic and translational science that spans many disciplines, and the implications could be profound. If we can manipulate self-renewal of stem cells, this could result in lucrative applications for directing self-renewal and differentiation for regenerative medicine, reversing degenerative diseases, as well as therapeutic interventions of cancer.

## Conflict of Interest Statement

The authors declare that the research was conducted in the absence of any commercial or financial relationships that could be construed as a potential conflict of interest.
